# A predictive model combining clinical characteristics and nutritional risk factors for overall survival after umbilical cord blood transplantation

**DOI:** 10.1186/s13287-023-03538-7

**Published:** 2023-10-23

**Authors:** Meijuan Tu, Aijie Huang, Lijuan Ning, Baolin Tang, Chunli Zhang, Guangyu Sun, Xiang Wan, Kaidi Song, Wen Yao, Ping Qiang, Yue Wu, Xiaoyu Zhu

**Affiliations:** 1 Department of Hematology, The First Affiliated Hospital of University of Science and Technology of China, Division of Life Sciences and Medicine, University of Science and Technology of China, Hefei, Anhui 230001 China; 2 Anhui Provincial Key Laboratory of Blood Research and Applications, Hefei, Anhui 230001 China; 3 Blood and Cell Therapy Institute, Division of Life Sciences and Medicine, University of Science and Technology of China, Hefei, Anhui 230001 China; 4 Department of Pharmacy, The First Affiliated Hospital of University of Science and Technology of China, Division of Life Sciences and Medicine, University of Science and Technology of China, Hefei, Anhui 230001 China; 5 Anhui Provincial Key Laboratory of Precision Pharmaceutical Preparations and Clinical Pharmacy, Hefei, Anhui 230001 China

**Keywords:** Predictive model, Umbilical cord blood transplantation, Nomogram, Nutritional risk factors

## Abstract

**Background:**

Umbilical cord blood transplantation (UCBT) is a curable therapy for hematological disease; however, the impact of nutritional status on UCBT outcomes remains controversial. To evaluate the joint effect of clinical characteristics and nutritional status on the prognosis of patients who underwent UCBT, we screened various factors to establish a predictive model of overall survival (OS) after UCBT.

**Methods:**

We performed an integrated clinical characteristic and nutritional risk factor analysis and established a predictive model that could be used to identify UCBT recipients with poor OS. Internal validation was performed by using the bootstrap method with 500 repetitions.

**Results:**

Four factors, including disease status, conditioning regimen, calf skinfold thickness and albumin level, were identified and used to develop a risk score for OS, which showed a positive predictive value of 84.0%. A high-risk score (≥ 2.225) was associated with inferior 3-year OS post-UCBT [67.5% (95% CI 51.1–79.4%), *P* = 0.001]. Then, we built a nomogram based on the four factors that showed good discrimination with a C-index of 0.833 (95% CI 0.743–0.922). The optimism-corrected C-index value of the bootstrapping was 0.804. Multivariate analysis suggested that a high calf skinfold thickness (≥ 20.5 mm) and a low albumin level (< 33.6 g/L) conferred poor disease-free survival (DFS).

**Conclusion:**

The predictive model combining clinical and nutritional factors could be used to predict OS in UCBT recipients, thereby promoting preemptive treatment.

## Introduction

Umbilical cord blood transplantation (UCBT) has been performed to treat hematological and nonhematological diseases for over 30 years, with the advantages of availability, fewer restrictions associated with human leukocyte antigen (HLA) matching, a low rate of relapse for patients with positive minimal residual disease (MRD) pretransplant and a low incidence of chronic graft-versus-host disease (GvHD) [1, 2]. However, the low total nucleated cell (TNC) and CD34^+^ cell doses in a single cord blood unit retrain the curative effect of UCBT, which may result in the occurrence of delayed engraftment, graft failure and infection that increases the risk of transplant-related mortality (TRM) [1–3].

Apart from the risk factors above [4, 5], HLA mismatch [5], cytomegalovirus (CMV) infection [4, 6], regimen-related toxicity [6], limited UCBT center experience [4] and malnutrition [7] have been associated with a high risk of mortality in UCBT recipients.

Hematopoietic stem cell transplantation (HSCT) carries nutritional risks resulting from high-dose chemotherapy alone or in combination with radiation therapy [8, 9]. In a prospective study, 21.2% of patients were at nutritional risk before HSCT according to Nutritional Risk Screening 2002 (NRS-2002), whereas the nutritional risk rate increased to 100% posttransplant [10]. Good nutritional status is beneficial for graft engraftment and immune reconstitution [11]. Furthermore, several studies have reported that disordered nutritional status during HSCT is related to inferior clinical outcomes as well as a higher complication rate during treatment, including reduced body mass index (BMI) [12], a decline in bone mineral density [13], lower serum albumin levels [14] and low vitamin D levels [13]. In recent years, a series of scales have been used to evaluate nutritional status or quality of life for predicting patient outcome, such as the Patient-Generated Subjective Global Assessment (PG-SGA) [15] and the Functional Assessment of Cancer Therapy-Bone Marrow Transplant (FACT-BMT) [16].

There is uncertainty regarding the extent to which nutritional parameters influence clinical outcomes in UCBT recipients. Thus, in this study, we assessed the joint effect of clinical characteristics and nutritional status on overall survival (OS) post-UCBT in adult recipients and screened risk factors to build a predictive model for identifying high-risk patients for early intervention.

## Materials and methods

### Patients

To effectively conduct questionnaire evaluation, we performed the questionnaire investigation with only youth and adults less than 65 years of age. Between September 2018 and December 2021, a total of 80 patients who underwent UCBT at the Department of Hematology, the First Affiliated Hospital of the University of Science and Technology of China (USTC), and received systemic nutritional evaluation were included in this study. The median age of the patients was 31 years (range 17–61 years), and 46.3% (37/80) were female.

All surviving patients were followed up from the date of transplantation until June 30, 2022, and the median follow-up time was 719.0 days (range 54–1262 days). The procedures were approved by the Ethics Committee of the First Affiliated Hospital of USTC (Approval number, 2022-RE-253). Patients or guardians provided informed consent before transplantation and for the use of data for research in accordance with the Declaration of Helsinki.

### Transplant protocols

The UCBT protocols were previously reported [17, 18]. The myeloablative conditioning (MAC) regimen was performed in 86.2% (69/80) of the patients, and the other 11 patients were treated with a reduced intensity conditioning (RIC) regimen. All patients were given cyclosporine (CsA) and mycophenolate mofetil (MMF) as GvHD prophylaxis after UCBT.

### Nutritional status and quality of life assessments

To evaluate the validity and quality of life questionnaire in patients who received UCBT and assess the effects on survival prognosis, a series of scales were evaluated in this study at day 30 after UCBT, including the Patient-Generated Subjective Global Assessment (PG-SGA) [19], the Exercise of Self-Care Agency (ESCA) [20], the General Self-Efficacy Scale (GSES) [21], the European Cancer Research and Treatment Organization Quality of Life Questionnaire-Cancer30 (EORTC-QLQ-C30) [22] and the Functional Assessment of Cancer Therapy-Bone Marrow Transplant (FACT-BMT) [23]. Body mass index (BMI), calf skinfold thickness, calf circumference, hand grip and albumin level were measured on the same day posttransplant, and BMI was also evaluated pretransplant. Patient weight was classified as underweight (BMI < 18.5 kg/m^2^), normal (BMI 18.5–24.9 kg/m^2^), and overweight (BMI ≥ 25 kg/m^2^) according to the World Health Organization [24].

### Definitions

OS was calculated from the date of transplantation until death or the last follow-up, and disease-free survival (DFS) was defined as the time from transplantation to either relapse or death of any cause. In the computation of the cumulative incidence of relapse (CIR) and nonrelapse mortality (NRM), relapse and death were considered competing events [25].

### Statistical analysis

For measurement data, the normality and outliers were explored by using histogram. Estimated probabilities for OS and DFS were calculated by using the Kaplan‒Meier method, and the significance levels associated with the survival curves were measured by using the log-rank test. The evaluation of CIR and NRM was performed by using Gray’s test. Univariate and multivariate analyses were evaluated using the Cox proportional hazard regression model. Factors with a *P* value < 0.1 in the univariate analysis were subjected to multivariate analysis. To validate the model, internal verification was performed using the bootstrapping method across 500 replicates, and the optimism-corrected C-index was calculated. The “rms” package of R version 4.2.1 software was used to prepare the nomogram and bootstrap. Receiver operating characteristic (ROC) curve analyses were performed, and areas under the curve (AUCs) were calculated with OS as the actual state variable. All statistical analyses were performed using SPSS 20.0. Figures were drawn by using R software (version 4.2.1) and GraphPad Prism 9. A *P* value less than 0.05 was considered statistically significant.

## Results

### Patients

#### General characteristics

A total of 80 patients who underwent single-unit UCBT at our transplantation center were analyzed retrospectively. Baseline patient demographic, disease and transplant characteristics are shown in Table 1. Thirty-seven patients were female, with a median age of 32 years (range 17–61 years), and the remaining 43 were male, with a median age of 30 years (range 17–49 years). Thirty-five patients (43.8%) were diagnosed with acute myeloid leukemia (AML), 20 (25.0%) with acute lymphoblastic leukemia (ALL), 13 (16.2%) with aplastic anemia (AA), 8 (10.0%) with myelodysplastic syndrome (MDS), and the remaining 4 (5.0%) had other diseases. A total of 68.7% (55/80) of the patients exhibited complete remission (CR) before UCBT. The mean infused total nucleated cell (TNC) and mean CD34^+^ cell values were 2.79 ± 3.35 × 10^7^/kg and 1.81 ± 1.24 × 10^5^/kg, respectively.

**Table 1:** Baseline characteristics of the patients treated with UCBT (n = 80)

Characteristic	
Age, median (range)	31 (17, 61)
Sex, *n* (%)	
Female	37 (46.3)
Male	43 (53.7)
Diagnosis, *n* (%)	
AML	35 (43.8)
ALL	20 (25.0)
AA	13 (16.2)
MDS	8 (10.0)
Other	4 (5.0)
Disease status prior to transplantation, *n* (%)	
PR/NR	25 (31.3)
CR	55 (68.7)
HLA compatibility (/10), *n* (%)	
≤ 6	25 (31.3)
7–8	44 (55.0)
≥ 9	11 (13.7)
Conditioning regimen, *n* (%)	
RIC	11 (13.8)
MAC	69 (86.2)
ABO incompatibility, *n* (%)	
Identical	29 (36.3)
Major	17 (21.3)
Minor	23 (28.7)
Bidirectional	11 (13.7)
Infused TNC, (mean ± SD) × 10^7^/kg	2.79 ± 3.35
Infused CD34^+^cells, (mean ± SD) × 10^5^/kg	1.81 ± 1.24
aGvHD, *n* (%)	
Grade 0–I	48 (60.0)
Grade II–IV	32 (40.0)
CMV infection posttransplant, *n* (%)	
With	61 (76.3)
Without	19 (23.7)
Pulmonary infection posttransplant, *n* (%)	
With	21 (26.3)
Without	59 (73.7)
PES, *n* (%)	
With	51 (63.7)
Without	29 (36.3)

*OS* overall survival, *DFS* disease-free survival, *UCBT* umbilical cord blood transplantation, *HR* hazard ratio, *CI* confidence interval, *AML* acute myeloid leukemia, *ALL* acute lymphoblastic leukemia, *AA* aplastic anemia, *MDS* myelodysplastic syndrome, *PR* partial remission, *NR* nonremission, *CR* complete remission, *HLA* human leukocyte antigen, *RIC* reduced intensity conditioning, *MAC* myeloablative conditioning, *TNC* total nucleated cell, *aGvHD* acute graft-versus-host disease, *CMV* cytomegalovirus, *PES* pre-engraftment syndrome

The data showed that 31.3% (25/80) of patients received lower high-resolution HLA compatibility (≤ 6/10) transplantation. The CMV infection and pulmonary infection occurred in 76.3% (61/80) and 26.3% (21/80) of patients, respectively. Furthermore, 51 patients suffered from pre-engraftment syndrome (PES).

### Nutritional status and quality of life evaluation

Table 2 provides the results of the patients’ nutritional status and quality of life evaluations. Fifty-one (63.8%) patients had a normal BMI, 11 (13.7%) had a low BMI, and 18 (22.5%) had a high BMI. A total of 78.8% (63/80) of the patients had weight loss calculated from the initial weight before UCBT to the weight at day 30 post-UCBT, and the mean weight loss ratio was 10.5% ± 6.9%. The median calf skinfold thickness, calf circumference and albumin level were 14.2 mm (range 4.5–30 mm), 27.5 cm (range 8.0–37.5 cm) and 33.3 g/L (range 23.6–48.2 g/L), respectively. According to the PG-SGA, 92.5% (74/80) of the patients were at high nutritional risk after UCBT (score ≥ 2), and 68.9% (51/74) of those patients were severely malnourished (score ≥ 9). The median scores on the ESCA, GSES, EORTC-QLQ C30 and FACT-BMT scales were 110 (range 23–150), 25 (range 10–58), 65 (range 43–93) and 81 (range 55–133), respectively.

**Table 2:** Nutritional status and quality of life evaluation of 80 patients

Evaluation index	
BMI before transplant, *n* (%)	
Underweight	11 (13.7)
Normal	51 (63.8)
Overweight	18 (22.5)
BMI at + 30 d posttransplant t, *n* (%)	
Overweight	10 (12.5)
Normal/Underweight	70 (87.5)
Ratio of BMI change, *n* (%)	
Reduced ≥ 8%	38 (47.5)
Increased/Reduced < 8%	42 (52.5)
Calf skinfold thickness, *n* (%)	
< 20.5 mm	61 (76.3)
≥ 20.5 mm	14 (17.5)
NA	5 (6.2)
Calf circumference, (mean ± SD) cm	26.76 ± 5.50
Hand grip, (mean ± SD)	21.66 ± 11.57
Albumin, g/L, *n* (%)	
< 33.6	42 (52.5)
≥ 33.6	38 (47.5)
PG-SGA, *n* (%)	
A (0–1, well-nourished)	1 (1.2)
B (2–8, suspected malnourished)	23 (28.8)
C (≥ 9, severe-malnourished)	51 (63.8)
NA	5 (6.2)
ESCA score, *n* (%)	
≥ 122.5	13 (16.3)
< 122.5	55 (687)
NA	12 (15.0)
GSES score, mean ± SD	26.22 ± 8.41
EORTC-QLQ C30 score, *n* (%)	
≥ 59.5	49 (61.3)
< 59.5	16 (20.0)
NA	15 (18.7)
FACT-BMT score, *n* (%)	
≥ 81	32 (40.0)
< 81	35 (43.8)
NA	13 (16.2)

*BMI* body mass index, *PG-SGA* patient-generated subjective global assessment, *ESCA* Exercise of Self-Care Agency, *GSES* General Self-Efficacy Scale, *EORTC-QLQ-C30* European Organization for Research and Treatment of Cancer Quality of Life Questionnaire, *FACT-BMT* Functional Assessment of Cancer Therapy-Bone Marrow Transplantation scale, *NA* not available

### Clinical outcomes

#### OS, DFS, aGvHD, CIR and NRM

**Fig. 1:**
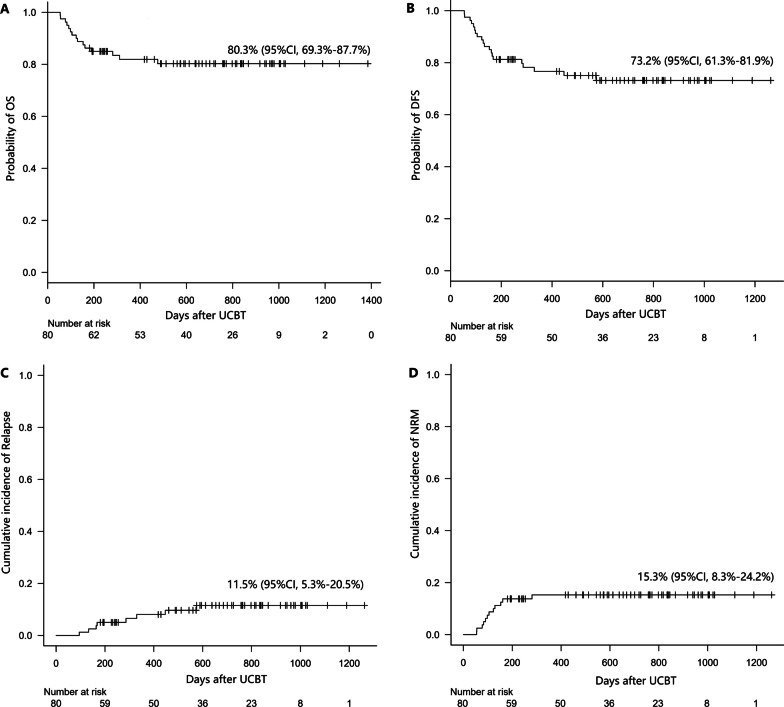
Survival of eighty UCBT recipients. **A** OS; **B** DFS; **C** CIR; **D** NRM

After UCBT, 36 patients developed aGvHD, including 32 patients with grade II-IV aGvHD and 23 patients with grade III-IV aGvHD. Overall, 8 patients (10%) relapsed after UCBT, and 12 patients died of TRM. The probability of 3-year OS was 80.3% (95% CI 69.3–87.7%), and the probability of 3-year DFS was 73.2% (95% CI 61.3–81.9%) (Fig. 1A, B). The cumulative incidences of 3-year CIR and 3-year NRM were 11.5% (95% CI 5.3–20.5%) and 15.3% (95% CI 8.3–24.2%), respectively (Fig. 1C, D).

### Risk factors for survival

Furthermore, we evaluated a series of parameters, including clinical characteristics, nutritional status and quality of life evaluation indexes, for a possible association with an increased risk of OS and DFS by using univariate Cox regression analysis, as shown in Table 3. The results suggested that diagnosis (AA vs. AML, HR: 4.373, 95% CI 1.169–16.359, *P* = 0.028), disease status before transplantation [partial remission (PR)/nonremission (NR) vs. CR, HR: 3.610, 95% CI 1.283–10.157, *P* = 0.015), conditioning regimen (RIC vs. MAC, HR: 3.971, 95% CI 1.351–11.672, *P* = 0.012), calf skinfold thickness (≥ 20.5 mm vs. < 20.5 mm, HR: 3.155, 95% CI 1.050–9.479, *P* = 0.041) and albumin level (< 33.6 g/L vs. ≥ 33.6 g/L, HR: 6.756, 95% CI 1.524–29.952, *P* = 0.012) were significantly associated with an increased risk of OS. PR/NR before transplantation [HR 2.635 (95% CI 1.095–6.342), *P* = 0.031], higher calf skinfold thickness [HR 2.691 (95% CI 1.016–7.128), *P* = 0.046], and low albumin level [HR 3.196 (95% CI 1.161–8.798), *P* = 0.025] were adverse prognostic factors for DFS. Subsequently, we performed multivariate Cox regression analysis for OS and DFS, and factors with a *P* value of ≤ 0.1 were included. In the multivariate analysis for OS, the PR/NR status pretransplantation had an HR of 7.948 (95% CI 1.405–44.963, *P* = 0.019), the RIC regimen had an HR of 12.707 (95% CI 1.041–155.049,* P* = 0.046), higher calf skinfold thickness had an HR of 6.940 (95% CI 1.699–28.345,* P* = 0.007) and low albumin level had an HR of 44.701 (95% CI 3.443–580.360, *P* = 0.004). The multivariate Cox regression analysis for DFS indicated that a high calf skinfold thickness and low albumin level were independent significant predictors (calf skinfold thickness: HR 3.485, 95% CI 1.180–10.289, *P* = 0.024; albumin: HR 5.612, 95% CI 1.705–18.474, *P* = 0.005).

**Table 3:** Univariate and multivariate analysis of OS in 80 patients treated with UCBT

Characteristic	OS						DFS					
	Univariate analysis			Multivariate analysis			Univariate analysis			Multivariate analysis		
	HR	95% CI	*P*	HR	95% CI	*P*	HR	95% CI	*P*	HR	95% CI	*P*
Age	1.021	0.976–1.067	0.369				1.006	0.966–1.047	0.782			
Sex, female vs. male	0.570	0.195–1.668	0.305				1.199	0.499–2.883	0.685			
Diagnosis												
AML			0.247			0.535			0.655			
ALL	1.480	0.330–6.643	0.609	2.895	0.545–15.383	0.212	1.473	0.464–4.672	0.511			
AA	4.373	1.169–16.359	**0.028**	0.389	0.026–5.903	0.496	2.501	0.791–7.907	0.118			
MDS	2.759	0.503–15.135	0.242	0.904	0.122–6.728	0.922	1.553	0.322–7.497	0.584			
Other	2.685	0.298–24.187	0.379	0.083	0–31.607	0.412	1.537	0.188–12.553	0.688			
Disease status prior to transplantation												
PR/NR vs. CR	3.610	1.283–10.157	**0.015**	7.948	1.405–44.963	**0.019**	2.635	1.095–6.342	**0.031**	2.408	0.860–6.741	0.094
HLA compatibility (/10)												
≤ 6			0.962						0.834			
7–8	0.855	0.280–2.615	0.784				1.332	0.469–3.782	0.590			
≥ 9	0.934	0.181–4.289	0.935				1.451	0.346–6.093	0.611			
Conditioning regimen												
RIC vs. MAC	3.971	1.351–11.672	**0.012**	12.707	1.041–155.049	**0.046**	2.665	0.965–7.355	**0.058**	3.186	0.874–11.616	0.079
ABO incompatibility												
Identical			0.821						0.251			
Major	1.544	0.471–5.064	0.473				1.981	0.742–5.288	0.172			
Minor	0.842	0.237–2.982	0.789				0.610	0.184–2.028	0.420			
Bidirectional	0.000	0-NA	0.978				0.000	0-NA	0.976			
Infused TNC, × 10^7^/kg	1.077	0.997–1.163	**0.058**	1.219	0.975–1.524	0.082	1.070	0.990–1.156	**0.086**	1.071	0.983–1.168	0.117
Infused CD34^+^cells, × 10^5^/kg	1.046	0.705–1.551	0.824				1.002	0.697–1.443	0.990			
aGvHD, grade II-IV vs. 0-I	1.919	0.695–5.299	0.208				1.351	0.559–3.262	0.504			
CMV, with vs. without	0.880	0.280–2.765	0.827				1.298	0.434–3.884	0.641			
Pulmonary infection, with vs. without	1.521	0.520–4.453	0.444				1.353	0.519–3.523	0.536			
PES, with vs. without	1.227	0.419–3.591	0.709				0.730	0.303–1.763	0.485			
BMI before transplant												
Underweight			0.668						0.589			
Normal	2.453	0.317–18.999	0.390				0.636	0.145–2.781	0.548			
Overweight	1.917	0.199–18.430	0.573				0.562	0.162–1.942	0.362			
BMI at + 30d posttransplant												
Overweight vs. normal/underweight	1.163	0.262–5.157	0.843				0.810	0.188–3.493	0.777			
Ratio of BMI change, reduced ≥ 8% vs. increased/reduced < 8%	2.335	0.798–6.836	0.122				1.400	0.580–3.380	0.454			
Calf skinfold thickness												
≥ 20.5 mm vs. < 20.5 mm	3.155	1.050–9.479	**0.041**	6.940	1.699–28.345	**0.007**	2.691	1.016–7.128	**0.046**	3.485	1.180–10.289	**0.024**
Calf circumference, cm	1.014	0.918–1.119	0.790				1.012	0.930–1.100	0.785			
Hand grip	0.969	0.917–1.024	0.265				0.968	0.923–1.015	0.175			
Albumin, < 33.6 g/L vs. ≥ 33.6 g/L	6.756	1.524–29.952	**0.012**	44.701	3.443–580.360	**0.004**	3.196	1.161–8.798	**0.025**	5.612	1.705–18.474	**0.005**
PG-SGA												
A			0.778						0.339			
B	6052.070	0–3.396E + 176	0.966				0.194	0.022–1.743	0.143			
C	9644.803	0–5.406E + 176	0.964				0.292	0.038–2.242	0.236			
ESCA, ≥ 122.5 vs. < 122.5	2.737	0.800–9.361	0.109				1.528	0.493–4.739	0.463			
GSES	1.014	0.949–1.083	0.687				0.997	0.940–1.058	0.928			
EORTC-QLQ-C30, ≥ 59.5 vs. < 59.5	33.388	0.091–12199.203	0.244				2.480	0.558–11.020	0.233			
FACT-BMT, ≥ 81 vs. < 81	1.105	0.296–4.119	0.882				1.144	0.396–3.306	0.804			

*Abbreviations*: Same as Tables 1 and 2

A *P* value less than 0.05 was considered statistically significant

### Development of a predictive model for OS

**Fig. 2:**
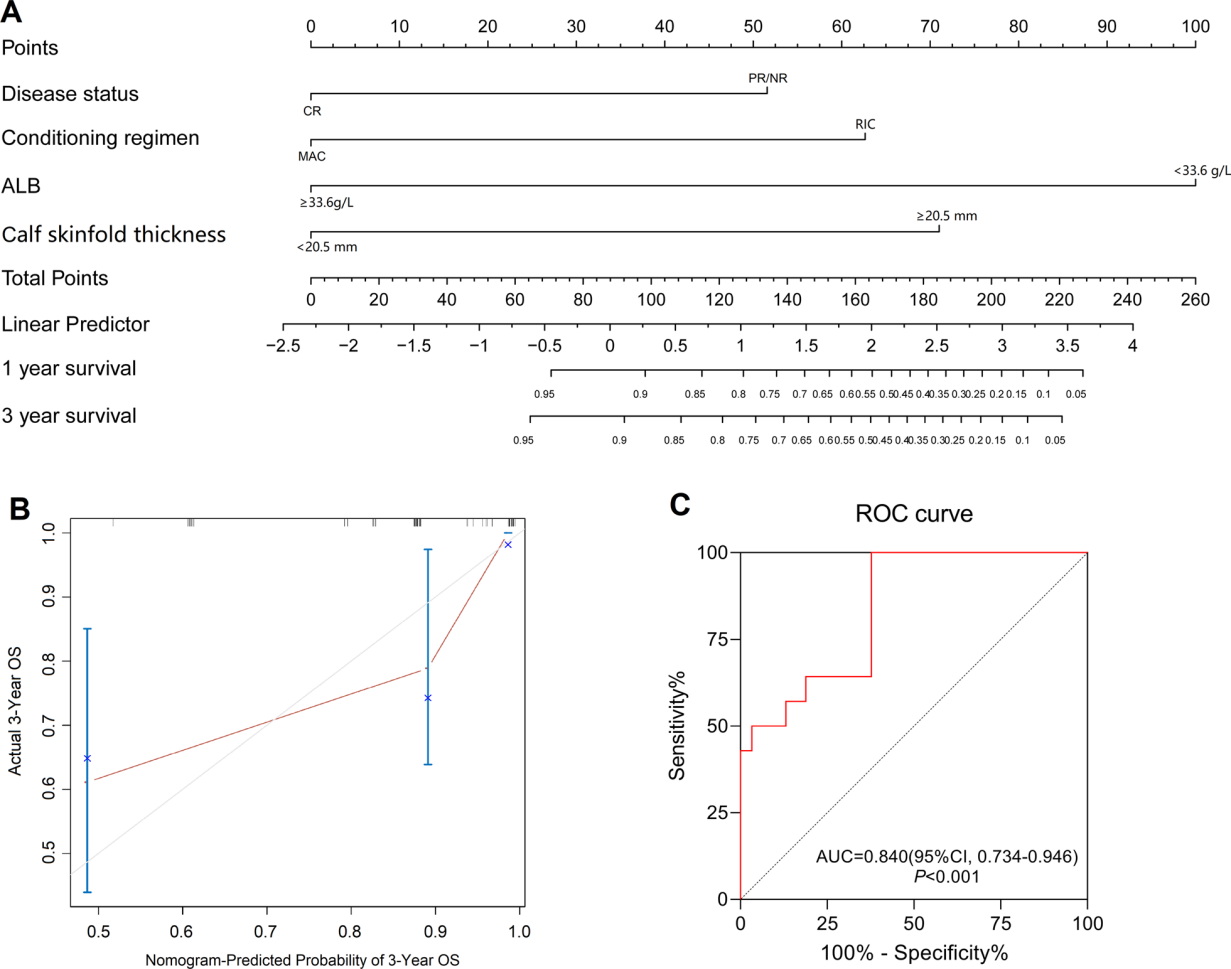
Nomogram for predicting the risk of OS. **A** Nomogram based on data from 80 patients for predicting the risk of OS; **B** The calibration curve showed the comparison between the predicted and actual 2-year OS in the internal verification; **C** ROC curve analysis of the ability of the four factors, namely disease status before UCBT, conditioning regimen, ALB and calf skinfold thickness, to predict OS. The area under the ROC curve was 0.840 (9% CI, 0734–0.948)

According to the multivariate regression analysis, disease status, conditioning regimen, calf skinfold thickness and albumin level were screened to construct a predictive model for OS (Fig. 2A). Furthermore, we calculated the risk score based on the individual expression levels of the four risk factors, where the risk score = 1.342 × v1 + 1.630 × v2 + 2.603 × v3 + 1.848 × v4 (Table 4). The time-dependent AUC was 0.840 (95% CI 0.734–0.946, *P* < 0.001, Fig. 2C), which suggested that the model for OS had considerable discriminative abilities.

**Table 4:** A panel of four factors with predictive value for OS

Factor	AUC of ROC curve	Regression weight
v1, Disease status	0.677 (0.519–0.835)	1.342
v2, Conditioning regimen	0.621 (0.448–0.793)	1.630
v3, ALB	0.710 (0.578–0.843)	2.603
v4, Calf skinfold thickness	0.605 (0.429–0.781)	1.848
The risk score of OS for each patient was calculated by using 4 factors according to the following equation: Risk score = 1.342 × v1 + 1.630 × v2 + 2.603 × v3 + 1.848 × v4		

**Fig. 3:**
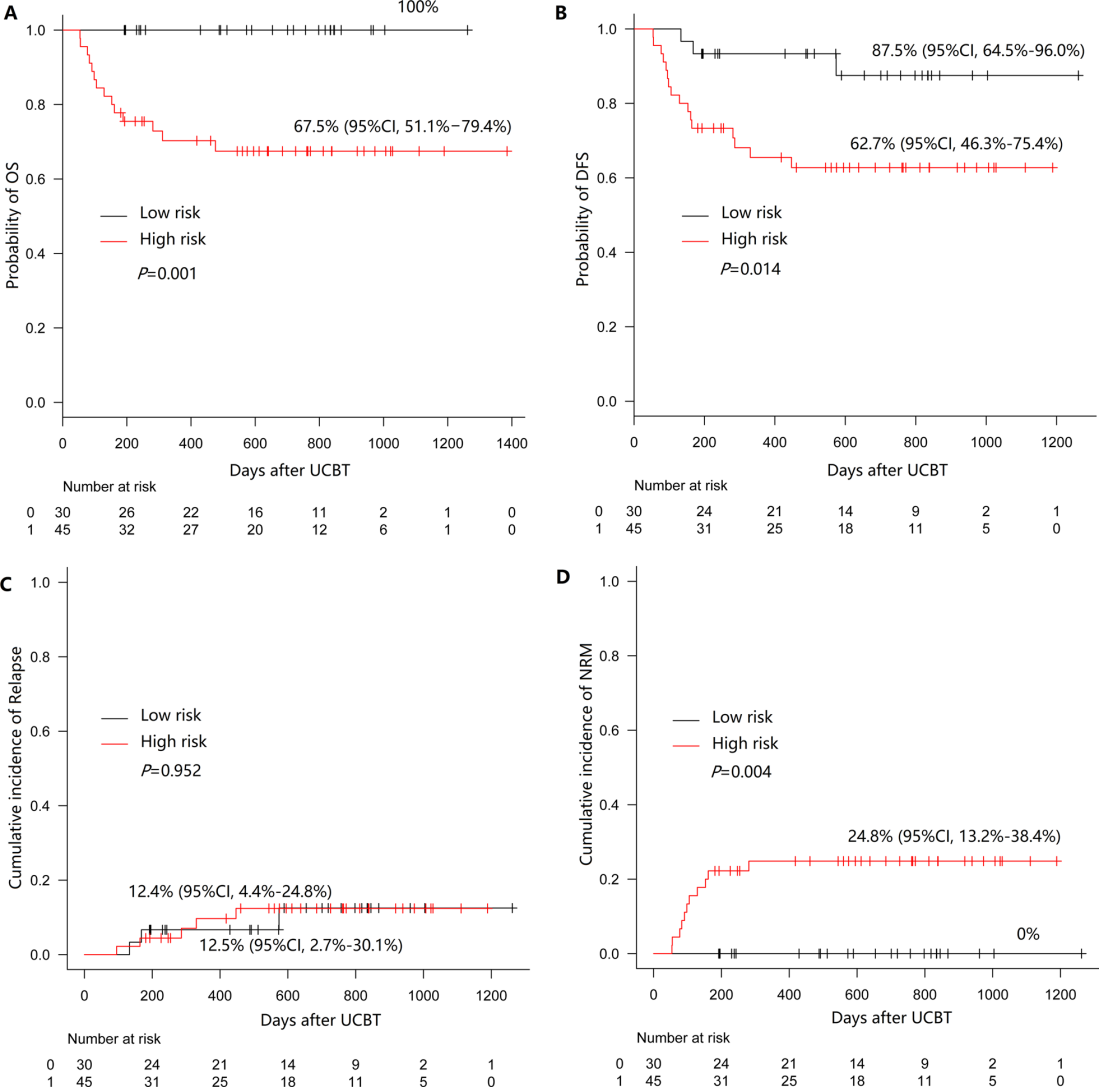
Survival outcomes according to the risk model for OS in patients who underwent UCBT (low risk: n = 30; high risk, HR: n = 45). **A** OS; **B** DFS; **C** CIR; **D** NRM

The optimal risk score cutoff was 2.225. The patients were divided into low-risk (score < 2.225, n = 30) and high-risk groups (score ≥ 2.225, n = 45) according to the cutoff value. The high-risk patients had poorer survival than the low-risk patients [3y-OS: 67.5% (95% CI 51.1–79.4%) vs. 100%, *P* = 0.001, Fig. 3A; 3y-DFS: 62.7% (95% CI 46.3–75.4%) vs. 87.5% (95% CI 64.5–96.0%), *P* = 0.014, Fig. 3B]. The rates of 3-year CIR were similar in the two groups [low risk: 12.5% (95% CI 2.7–30.1%), high risk: 12.4% (95% CI 4.4–24.8%), *P* = 0.952]. Compared with the patients in the low-risk group, those in the high-risk group had a higher NRM rate [24.8% (95% CI 13.2–38.4%) vs. 0%, *P* = 0.004, Fig. 3D].

Subsequently, we built a nomogram based on the panel of the model to evaluate its clinical application (Fig. 2A). The C-index value was 0.833 (95% CI 0.743–0.922). In the internal verification, the corrected C-index was 0.804, which indicated good concordance between the predicted and actual 3-year OS (Fig. 2B).

### Calf skinfold thickness for predicting disease-free-survival

**Fig. 4:**
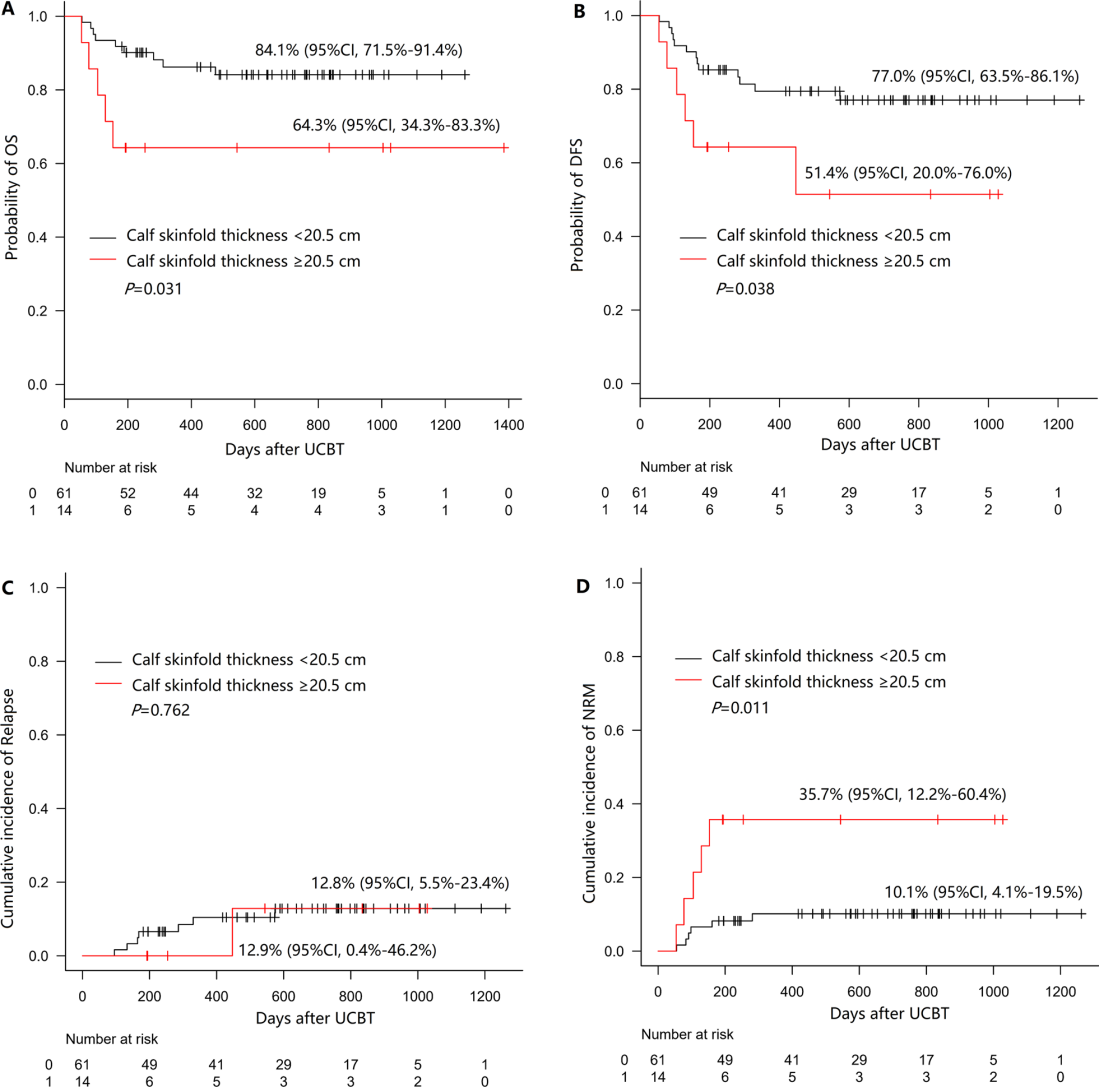
Survival outcomes according to the calf skinfold thickness (≥ 20.5 cm: n = 14; < 20.5 cm: n = 61). The results are shown for **A** OS, **B** DFS, **C** CIR, and **D** NRM

The cutoff point of the calf skinfold thickness was 20.5, as calculated by ROC analysis, and the patients were divided into a low calf skinfold thickness group (< 20.5, n = 61) and a high calf skinfold thickness (≥ 20.5, n = 14). The results of multivariate analysis suggested that high calf skinfold thickness was an independent risk factor for DFS. Patients with high calf skinfold thickness had inferior OS, DFS and NRM than those with low calf skinfold thickness [3y-OS: 64.3% (95% CI 34.3–83.3%) vs. 84.1% (95% CI 71.5–91.4%, *P* = 0.031, Fig. 4A; 3y-DFS: 51.4% (95% CI 20.0–76.0%) vs. 77.0% (95% CI 63.5–86.1%), *P* = 0.038, Fig. 4B; 3y-NRM: 35.7% (95% CI 12.2–60.4%) vs. 10.1% (95% CI 4.1–19.5%), *P* = 0.011, Fig. 4D]. The CIR rates between the two groups showed no differences (*P* = 0.762, Fig. 4C).

## Discussion

Chemotherapy and radiation therapy not only damage tumor cells but also significantly impair proliferative cells, such as colonic epithelial cells and lymphocytes, which may cause metabolic disorders and undernutrition [11]. Our results showed that 13.75% (11/80) of patients had a low BMI of < 18.5 kg/m^2^ before UCBT, and the proportion increased to 27.5% (22/80) at day 30 post-UCBT. Moreover, 47.5% (38/80) of patients experienced weight loss of > 8% of their initial weight before transplantation. These data suggested that patients were at high nutritional risk during transplantation, which was similar to a previous report by Peng Liu et al. [10]. They enrolled 170 allo-HSCT recipients and found that 50.46% of the patients had weight loss of more than 10% post-HSCT.

As in previous reports, various factors influence the outcome of HSCT [4, 5, 7, 26]. In addition to clinical characteristics, nutritional status plays an important role in patient survival [7, 11]. Thus, in this study, we analyzed risk factors affecting survival by combining clinical factors (such as diagnosis and disease status), nutritional and physical functional assessment indicators, including laboratory tests (albumin level), physical measures (BMI, calf skinfold thickness, calf circumference and hand grip) and scales (PG-SGA, ESCA, GSES, EORTC-QLQ C30 and FACT-BMT). In multivariate analysis, RIC regimen, PR/NR status before transplantation, calf skinfold thickness and albumin level were independent risk factors for OS. A higher calf skinfold thickness and lower albumin level were related to poorer DFS. Then, a risk model for OS was established based on the four factors. The patients with high-risk scores (≥ 2.225) had poorer survival than those with low-risk scores [3-year OS: 67.5% (95% CI 51.1–79.4%), *P* = 0.001; 3-year DFS: 62.7% (95% CI 46.3–75.4%), *P* = 0.014] (Fig. 3).

Although many indicators were used to evaluate nutritional status in UCBT recipients, the results showed that only calf skinfold thickness and albumin level were related to survival. In our study, the above scales seem useless for predicting outcomes in patients undergoing UCBT, which suggests that laboratory indictors and physical measurements are more important than subjective scales for predicting the survival of UCBT recipients. Although we choose the same time-point of survey, the results of scales may also have bias due to differences in education and the physical and mental state of patients. Our results could simplify the evaluation type of scale and provide a practical direction for clinical work.

In previous studies, scholars reported that low albumin level was related with inflammation and poor prognosis [27–29]. Our data showed that the median level of albumin was 33.3 g/L (range 23.6–48.2 g/L) at 30 days post-UCBT, which was lower than the normal limits in our hospital (40–55 g/L). Patients had a reduced albumin levels post-transplantation, which was similar to the study reported by Stephanie et al. [30]. These patients with lower serum albumin (< 33.6 g/L) had inferior OS and DFS [OS: 67.4% (95% CI, 50.3–79.7%) vs. 94.7% (95% CI, 80.6–98.7%), *P* = 0.004; DFS: 62.3% (95% CI, 45.2–75.5%) vs. 85.4% (95% CI, 67.8–93.8%) *P* = 0.018]. Some studies identified serum albumin as a predictive marker for severe aGvHD in adult and pediatric patients post-HSCT [28, 31, 32]. The decline of albumin level may be due to impaired synthesis and increased catabolism caused by inflammation and gut damage [27, 31]. In our study, patients with lower albumin level had higher aGvHD rates; among them, 54.8% (23/42) patients developed grade II-IV aGvHD. Twenty of 23 (87.0%) aGvHD patients had gastrointestinal tract involvement.

Skinfold thickness reflects body fat level. Researchers usually assess fat mass by using the skinfold thickness at 5 to 9 body sites, such as the triceps, biceps, abdominal and calf skinfold thicknesses [10, 33]. In our center, UCBT recipients underwent insertion of central venous catheters on both upper arms. Therefore, we measured the circumference and skinfold thickness of the calf instead of the arm. In the present study, 14 patients had a higher calf skinfold thickness (≥ 20.5 mm), and among them, 57.1% (8/14) had a weight loss of > 8%. The significance of calf skinfold thickness on transplantation outcomes has not been reported. We observed that patients with a high calf skinfold thickness had an 8.289-fold risk of inferior OS than patients with a lower skinfold thickness (*P* = 0.011) and a 3.723-fold risk of poorer DFS (*P* = 0.016). Moreover, the measurement of calf skinfold thickness is a noninvasive and convenient examination that can be closely monitored in UCBT recipients.

The role of BMI in predicting outcomes is controversial. In a retrospective analysis of 2503 patients who underwent HSCT, the authors found that both underweight and obese patients had an increased NRM compared with normal-weight HSCT recipients [34]. Prasad and collaborators [35] conducted a randomized controlled phase-3 open-label trial to evaluate the effect of arm anthropometry on nutritional assessment, and the study showed that the addition of arm anthropometry (mid-upper arm circumference and triceps skinfold thickness) to BMI increased the sensitivity of nutritional evaluation. However, in our study, BMI and the decline in BMI post-HSCT showed no significant effect on OS and DFS, which was supported by other studies [36, 37].

The risk score generated from the 4 factors we identified could be used to predict OS with an AUC of 0.840 (Fig. 2C). Furthermore, based on these factors, we developed a nomogram for clinical application to help identify high-risk patients with inferior OS. Calibration plots of the nomograms showed that the nomograms performed well compared with an ideal model. By using this model, we can distinguish high-risk patients and provide early nutritional treatment.

To the best of our knowledge, this is the first study to evaluate the survival of UCBT patients by integrating clinical factors and various nutritional indexes and to build a risk model to identify high-risk patients and facilitate early interventions. However, there were some limitations in this study, such as a small sample size, a lack of a validation set, and the absence of detailed food consumption. Although internal validation by the bootstrap method with a corrected c-index of 0.804 was performed in our study, external validation is still important; thus, a multicenter clinical trial to validate our predictive model is necessary in future. Additionally, we did not investigate the specific mechanisms underlying the association between nutritional factors and UCBT outcomes, which warrants further exploration in future research.

## Conclusion

In conclusion, the predictive model combining clinical and nutritional factors could be used to predict survival and stratified the survival of different groups in UCBT recipients, which may promote preemptive treatment.

## Data Availability

The data that support the findings of this study are available from the corresponding author upon reason-ablerequest.

## Abbreviations

UCBT: Umbilical cord blood transplantation
OS: Overall survival
DFS: Disease-free survival
HLA: Human leukocyte antigen
MRD: Minimal residual disease
GvHD: Graft-versus-host disease
TNC: Total nucleated cell
TRM: Transplant-related mortality
CMV: Cytomegalovirus
HSCT: Hematopoietic stem cell transplantation
NRS-2002: Nutritional risk screening 2002
BMI: Body mass index
PG-SGA: Patient-Generated Subjective Global Assessment
FACT-BMT: Functional Assessment of Cancer Therapy-Bone Marrow Transplant
MAC: Myeloablative conditioning
RIC: Reduced intensity conditioning
CsA: Cyclosporine
MMF: Mycophenolate mofetil
ESCA: Exercise of self-care agency
GSES: General self-efficacy scale
EORTC-QLQ-C30: European Cancer Research and Treatment Organization Quality of Life Questionnaire-Cancer30
CIR: Cumulative incidence of relapse
NRM: Nonrelapse mortality
ROC: Receiver operating characteristic
AUC: Areas under the curve
ALL: Acute lymphoblastic leukemia
AA: Aplastic anemia
MDS: Myelodysplastic syndrome
CR: Complete remission

## References

[1] Zhu X, Tang B, Sun Z (2021). Umbilical cord blood transplantation: still growing and improving. Stem Cells Transl Med.

[2] Mayani H, Wagner JE, Broxmeyer HE (2020). Cord blood research, banking, and transplantation: achievements, challenges, and perspectives. Bone Marrow Transplant.

[3] Kindwall-Keller TL, Ballen KK (2020). Umbilical cord blood: The promise and the uncertainty. Stem Cells Transl Med.

[4] Cohen YC, Scaradavou A, Stevens CE, Rubinstein P, Gluckman E, Rocha V, Horowitz MM, Eapen M, Nagler A, Shpall EJ (2011). Factors affecting mortality following myeloablative cord blood transplantation in adults: a pooled analysis of three international registries. Bone Marrow Transplant.

[5] Kanda J, Hayashi H, Ruggeri A, Kimura F, Volt F, Takahashi S, Labopin M, Kako S, Tozatto-Maio K, Yano S (2020). Prognostic factors for adult single cord blood transplantation among European and Japanese populations: the Eurocord/ALWP-EBMT and JSHCT/JDCHCT collaborative study. Leukemia.

[6] Spees LP, Martin PL, Kurtzberg J, Stokhuyzen A, McGill L, Prasad VK, Driscoll TA, Parikh SH, Page KM, Vinesett R (2019). Reduction in mortality after umbilical cord blood transplantation in children over a 20-year period (1995–2014). Biol Blood Marrow Transplant.

[7] Kerby EH, Li Y, Getz KD, Smith EC, Smith LT, Bunin NJ, Seif AE: Nutritional risk factors predict severe acute graft-versus-host disease and early mortality in pediatric allogeneic hematopoietic stem cell transplantation. Pediatr Blood Cancer 2018;65. 10.1002/pbc.26853 29080380

[8] McMillen KK, Coghlin-Dickson T, Adintori PA (2021). Optimization of nutrition support practices early after hematopoietic cell transplantation. Bone Marrow Transplant.

[9] Wang D, Sun Z, Zhu X, Zheng X, Zhou Y, Lu Y, Yan P, Wang H, Liu H, Jin J (2022). GARP-mediated active TGF-beta1 induces bone marrow NK cell dysfunction in AML patients with early relapse post-allo-HSCT. Blood.

[10] Liu P, Wang B, Yan X, Cai J, Wang Y (2016). Comprehensive evaluation of nutritional status before and after hematopoietic stem cell transplantation in 170 patients with hematological diseases. Chin J Cancer Res.

[11] Martin-Salces M, de Paz R, Canales MA, Mesejo A, Hernandez-Navarro F (2008). Nutritional recommendations in hematopoietic stem cell transplantation. Nutrition.

[12] Pereira AZ, de Almeida-Pitito B, Eugenio GC, Ruscitto do Prado R, Silva CC, Hamerschlak N (2021). Impact of obesity and visceral fat on mortality in hematopoietic stem cell transplantation. JPEN J Parenter Enteral Nutr.

[13] Campos DJ, Boguszewski CL, Funke VA, Bonfim CM, Kulak CA, Pasquini R, Borba VZ (2014). Bone mineral density, vitamin D, and nutritional status of children submitted to hematopoietic stem cell transplantation. Nutrition.

[14] Goussetis E, Paisiou A, Kitra V, Peristeri I, Vessalas G, Stefanaki K, Panayotou I, Giamaiou K, Kontou E, Kitzoni M (2011). Acute gastrointestinal graft-versus-host disease in pediatric patients: serum albumin on day 5 from initiation of therapy correlates with nonrelapse mortality and overall survival. Biol Blood Marrow Transplant.

[15] Wiegert EVM, Padilha PC, Peres WAF (2017). Performance of patient-generated subjective global assessment (PG-SGA) in patients with advanced cancer in palliative care. Nutr Clin Pract.

[16] Mishra A, Pidala J, Thapa R, Betts BC, Fernandez H, Locke FL, Nishihori T, Perez L, Wang X, Anasetti C (2021). Objective and subjective physical function in allogeneic hematopoietic stem cell transplant recipients. Bone Marrow Transplant.

[17] Wu Y, Zhang Z, Tu M, Pan T, Ding P, Tang B, Wan X, Yao W, Song K, Sun G (2022). Poor survival and prediction of prolonged isolated thrombocytopenia post umbilical cord blood transplantation in patients with hematological malignancies. Hematol Oncol.

[18] Sun Z, Liu H, Luo C, Geng L, Zheng C, Tang B, Zhu X, Tong J, Wang X, Ding K (2018). Better outcomes of modified myeloablative conditioning without antithymocyte globulin versus myeloablative conditioning in cord blood transplantation for hematological malignancies: a retrospective (development) and a prospective (validation) study. Int J Cancer.

[19] Erickson N, Storck LJ, Kolm A, Norman K, Fey T, Schiffler V, Ottery FD, Jager-Wittenaar H (2019). Tri-country translation, cultural adaptation, and validity confirmation of the scored patient-generated subjective global assessment. Support Care Cancer.

[20] Kearney BY, Fleischer BJ (1979). Development of an instrument to measure exercise of self-care agency. Res Nurs Health.

[21] Wang J, Yin Y, Li Y, Yue X, Qi X, Sun M (2021). The effects of solution-focused nursing on leukemia chemotherapy patients' moods, cancer-related fatigue, coping styles, self-efficacy, and quality of life. Am J Transl Res.

[22] Aaronson NK, Ahmedzai S, Bergman B, Bullinger M, Cull A, Duez NJ, Filiberti A, Flechtner H, Fleishman SB, de Haes JC (1993). The European Organization for Research and Treatment of Cancer QLQ-C30: a quality-of-life instrument for use in international clinical trials in oncology. J Natl Cancer Inst.

[23] McQuellon RP, Russell GB, Cella DF, Craven BL, Brady M, Bonomi A, Hurd DD (1997). Quality of life measurement in bone marrow transplantation: development of the Functional Assessment of Cancer Therapy-Bone Marrow Transplant (FACT-BMT) scale. Bone Marrow Transplant.

[24] Obesity: preventing and managing the global epidemic. Report of a WHO consultation. World Health Organ Tech Rep Ser 2000;894:i-xii, 1–253. 11234459

[25] Huang A, Chen Q, Fei Y, Wang Z, Ni X, Gao L, Chen L, Chen J, Zhang W, Yang J (2021). Dynamic prediction of relapse in patients with acute leukemias after allogeneic transplantation: Joint model for minimal residual disease. Int J Lab Hematol.

[26] Ruggeri A, Michel G, Dalle JH, Caniglia M, Locatelli F, Campos A, de Heredia CD, Mohty M, Hurtado JM, Bierings M (2012). Impact of pretransplant minimal residual disease after cord blood transplantation for childhood acute lymphoblastic leukemia in remission: an Eurocord PDWP-EBMT analysis. Leukemia.

[27] Don BR, Kaysen G (2004). Serum albumin: relationship to inflammation and nutrition. Semin Dial.

[28] Kharfan-Dabaja MA, Sheets K, Kumar A, Murthy HS, Nishihori T, Tsalatsanis A, Mina A, Mathews J, Ayala E, Chavez J (2018). Hypoalbuminaemia segregates different prognostic subgroups within the refined standard risk acute graft-versus-host disease score. Br J Haematol.

[29] Artz AS, Logan B, Zhu X, Akpek G, Bufarull RM, Gupta V, Lazarus HM, Litzow M, Loren A, Majhail NS (2016). The prognostic value of serum C-reactive protein, ferritin, and albumin prior to allogeneic transplantation for acute myeloid leukemia and myelodysplastic syndromes. Haematologica.

[30] Szovati S, Morrison CF, Couch SC (2023). Nutritional status of allogeneic hematopoietic stem cell transplant recipients and post-transplant outcomes. Nutr Cancer.

[31] Takahashi N, Mochizuki K, Sano H, Kobayashi S, Ohara Y, Ikeda K, Ohto H, Kikuta A (2021). Decline of serum albumin precedes severe acute GVHD after haploidentical HSCT. Pediatr Int.

[32] Rashidi A, DiPersio JF, Westervelt P, Abboud CN, Schroeder MA, Cashen AF, Pusic I, Romee R (2016). Peritransplant serum albumin decline predicts subsequent severe acute graft-versus-host disease after mucotoxic myeloablative conditioning. Biol Blood Marrow Transplant.

[33] Kryst L, Zeglen M, Artymiak P, Kowal M, Woronkowicz A (2022). The impact of lifestyle and socioeconomic parameters on body fat level in early childhood. J Biosoc Sci.

[34] Doney K, McMillen K, Buono L, Deeg HJ, Gooley T (2019). Impact of body mass index on outcomes of hematopoietic stem cell transplantation in adults. Biol Blood Marrow Transplant.

[35] Prasad M, Ladas EJ, Barr R (2022). Addition of arm anthropometry to body mass index for age, but not serum albumin, improves the accuracy of the nutritional assessment in severely and moderately malnourished children with cancer. Pediatr Blood Cancer.

[36] Hadjibabaie M, Tabeefar H, Alimoghaddam K, Iravani M, Eslami K, Honarmand H, Javadi MR, Khatami F, Ashouri A, Ghavamzadeh A (2012). The relationship between body mass index and outcomes in leukemic patients undergoing allogeneic hematopoietic stem cell transplantation. Clin Transplant.

[37] Aplenc R, Zhang MJ, Sung L, Zhu X, Ho VT, Cooke K, Dvorak C, Hale G, Isola LM, Lazarus HM (2014). Effect of body mass in children with hematologic malignancies undergoing allogeneic bone marrow transplantation. Blood.

